# Detection of *Cryptosporidium parvum* Oocysts on Fresh Produce Using DNA Aptamers

**DOI:** 10.1371/journal.pone.0137455

**Published:** 2015-09-03

**Authors:** Asma Iqbal, Mahmoud Labib, Darija Muharemagic, Syed Sattar, Brent R. Dixon, Maxim V. Berezovski

**Affiliations:** 1 Bureau of Microbial Hazards, Food Directorate, Health Canada, Ottawa, Ontario, Canada; 2 Department of Chemistry and Biomolecular Sciences, University of Ottawa, Ottawa, Ontario, Canada; 3 Department of Biochemistry, Microbiology and Immunology, Faculty of Medicine, University of Ottawa, Ottawa, Ontario, Canada; University of Houston, UNITED STATES

## Abstract

There are currently no standard methods for the detection of *Cryptosporidium* spp., or other protozoan parasites, in foods, and existing methods are often inadequate, with low and variable recovery efficiencies. Food testing is difficult due to the low concentrations of parasites, the difficulty in eluting parasites from some foods, the lack of enrichment methods, and the presence of PCR inhibitors. The main objectives of the present study were to obtain DNA aptamers binding to the oocyst wall of *C*. *parvum*, and to use the aptamers to detect the presence of this parasite in foods. DNA aptamers were selected against *C*. *parvum* oocysts using SELEX (Systematic Evolution of Ligands by EXponential enrichment). Ten rounds of selection led to the discovery of 14 aptamer clones with high affinities for *C*. *parvum* oocysts. For detecting parasite-bound aptamers, a simple electrochemical sensor was employed, which used a gold nanoparticle-modified screen-printed carbon electrode. This aptasensor was fabricated by self-assembling a hybrid of a thiolated ssDNA primer and the anti- *C*. *parvum* aptamer. Square wave voltammetry was employed to quantitate *C*. *parvum* in the range of 150 to 800 oocysts, with a detection limit of approximately 100 oocysts. The high sensitivity and specificity of the developed aptasensor suggests that this novel method is very promising for the detection and identification of *C*. *parvum* oocysts on spiked fresh fruits, as compared to conventional methods such as microscopy and PCR.

## Introduction

*Cryptosporidium* spp. is a common intestinal protozoan parasite occurring in humans and many animal species world-wide. In recent years, cryptosporidiosis has emerged as a global public health problem and this parasite is now considered to be a common cause of gastroenteritis in immunocompetent individuals, and of severe illness in immunocompromised individuals. There are currently 26 valid species of *Cryptosporidium*, and greater than 40 distinct genotypes [1], however, approximately 90% of reported human infections involve either *C*. *hominis*, which is found primarily in humans, or *C*. *parvum*, which is an important zoonotic species.

The infectious stage of *Cryptosporidium* spp., known as oocysts, are shed with the feces of a host and are immediately infective to subsequent hosts. Routes of transmission of cryptosporidiosis include waterborne, person-to-person (i.e., the fecal-oral route), zoonotic and foodborne [2]. Although there is considerable overlap amongst these routes of transmission, water is numerically the most important mode of transmission, with numerous outbreaks having occurred world-wide as a result of oocyst contamination of drinking water and recreational water [3]. Direct person-to-person transmission may occur following the ingestion of oocysts in fecal matter, and is associated with poor hygiene. In the case of zoonotic species of *Cryptosporidium*, such as *C*. *parvum*, calves, rodents, puppies, kittens, and many other animals serve as important reservoir hosts in zoonotic transmission [2].

Approximately 8% of domestically acquired cases of cryptosporidiosis in the USA are foodborne [4]. Fresh produce contaminated with *Cryptosporidium* oocysts is likely the most common source of foodborne infection as it is often consumed without any processing. Furthermore, there are numerous possible sources of contamination of fresh produce. For example, the irrigation of crops with fecally-contaminated water, and the use of contaminated water to mix pesticides or wash produce, have been identified as important sources of contamination. In the case of zoonotic species and genotypes, livestock and other domestic and wild animals may contaminate produce, either through direct contact or through the application of manure to crop lands as fertilizer [5]. Direct contamination of produce from farm workers or food handlers who are infected, or who are caring for infected individuals, may occur during harvesting, packaging, transportation or food preparation, and is largely associated with poor personal hygiene, namely insufficient hand washing.

Foodborne infections with *Cryptosporidium* spp., and other parasites, are of increasing concern in developed countries around the world due to a variety of factors such as the global nature of the food trade, international travel, the increased number of immunocompromised and other susceptible individuals, and changes in consumer habits, particularly the consumption of more raw and undercooked foods [6]. Numerous foodborne cases and outbreaks of illness due to infections with *Cryptosporidium* spp. have, in fact, been reported in developed countries in recent years [7]. While a very large number of surveillance studies have been done on the prevalence of *Cryptosporidium* spp. on fresh produce in developing countries world-wide, there are very few data available from developed regions of the world [7]. While this is likely due, mainly, to a lack of awareness of the issue of foodborne transmission of *Cryptosporidium* spp., it is also due, in part, to the lack of standardized methods available for testing. In clinical samples, the detection of *Cryptosporidium* spp. is often based on an initial fecal flotation to concentrate the oocysts, followed by microscopical examination of a direct fecal smear, or by modified acid-fast staining [8]. Although microscopy is the ‘gold standard’ for detecting enteric parasites, progress has been made in the past 15 years in developing and validating alternative diagnostic tests, including immunofluorescence microscopy using labeled monoclonal antibodies, and the polymerase chain reaction (PCR), both of which can offer increased sensitivity over conventional microscopy [9]. These methods have also been very useful in testing food samples. There are, however, many hurdles faced in testing food samples. These include, most notably, the low concentrations of parasites in foods, the difficulty in eluting parasites from some foods, the lack of enrichment methods, and the presence of PCR inhibitors. It is imperative, therefore, that parasite concentration and purification procedures are first performed to increase the likelihood of detecting any parasites that may be present in a food sample. Procedures such as filtration, centrifugation, and immunomagnetic separation, alone or in combination, may help to concentrate and purify the infectious stages of parasites from food debris and PCR inhibitors [6].

Molecular methods, relying on the high specificity of DNA hybridization/recognition reactions, hold great promise for environmental monitoring of *Cryptosporidium* spp. Recent reports have demonstrated the ability of electrochemical sensors to detect a variety of biomolecules, including proteins, carbohydrates, glycoproteins, nucleic acids, and microorganisms, with high sensitivity and excellent reproducibility [10]. Electrochemical detection is economic compared to other methods and more convenient for on-field applications because they can be miniaturized and do not require expensive optical instruments. Furthermore, modern eletcrochemical detectors allow simultaneous analysis of many samples in very short analysis time.

**Fig 1:**
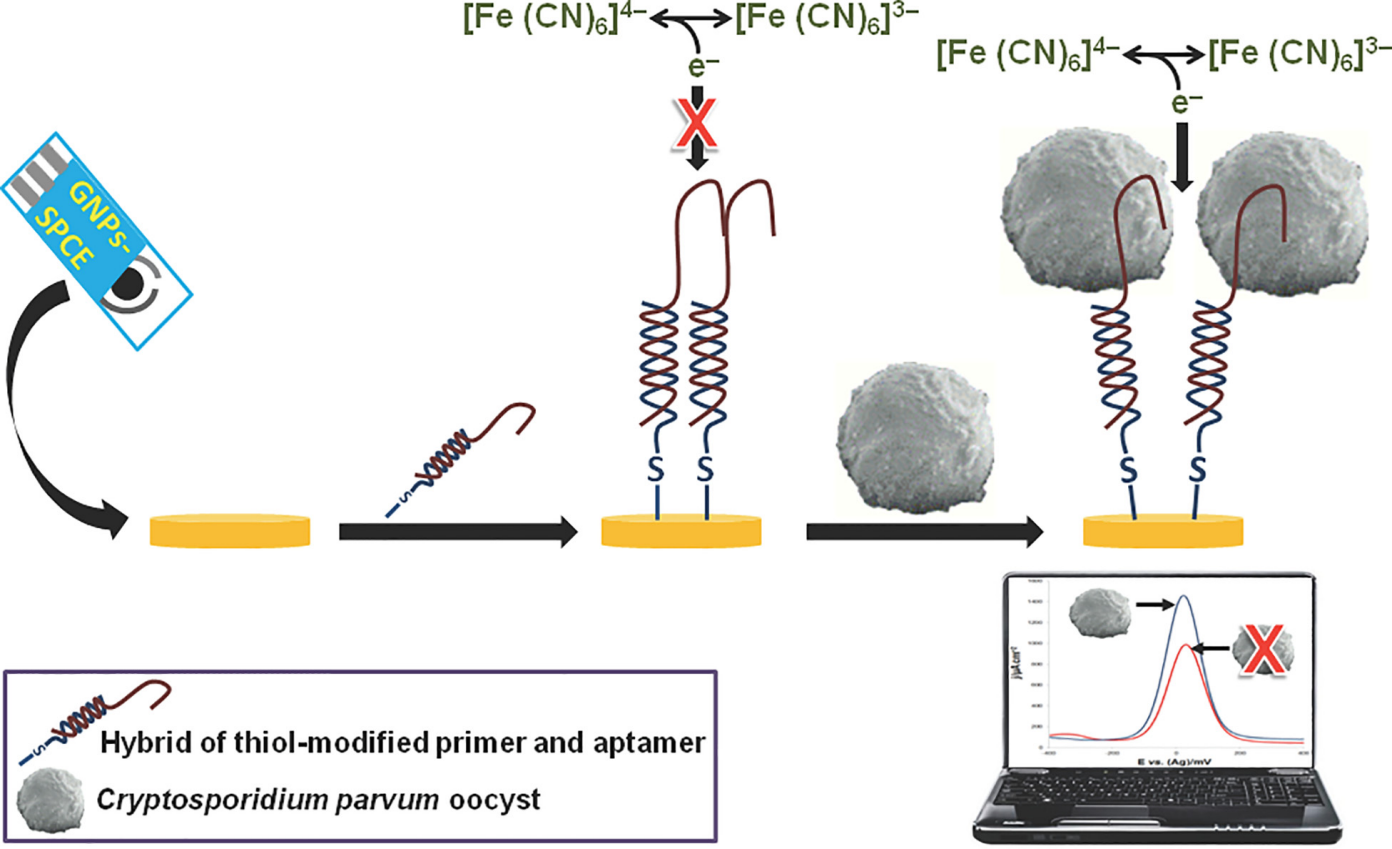
Schematic representation of an electrochemical detection protocol adopted for this study. A hybrid of a thiol-modified primer and aptamer was self-assembled onto a gold nanoparticles-modified screen-printed carbon electrode (GNPs-SPCE). Binding of the *Cryptosporidium parvum* oocyst to the immobilized aptamer causes an increase in the redox current, measured by square wave voltammetry.

The present study describes a novel aptamer-based electrochemical biosensor for detecting *C*. *parvum* oocysts on fresh produce. Aptamers are synthetic nucleic acids that fold into unique three-dimensional conformations capable of binding a target with remarkable affinity and specificity [11, 12]. For the present study, we selected several aptamers specific to *C*. *parvum* oocysts, sequenced them, and evaluated the selected aptamer sequences using an electrochemical aptasensor to estimate the binding strength of the selected aptamers, and to quantitate the oocysts. As shown in Fig 1, the sensing interface of the biosensor is based on the formation of a self-assembled monolayer of a thiolated-DNA capture probe and an oocyst-specific DNA aptamer onto a gold nanoparticle-modified screen-printed carbon electrode. Square wave voltammetry (SWV) was adopted for analysis as an effective and rapid technique with well-established advantages, including low detection limits and good discrimination against background currents.

## Materials and Methods

### Aptamer Selection

Aptamers were selected from a synthetic single-stranded (ss) DNA library (Integrated DNA Technologies, CA, USA) which consisted of a randomized region of 40 nucleotides (N40) flanked by two constant primer-hybridization sites, 5′-CTC CTC TGA CTG TAA CCA CG N40 GC ATA GGT AGT CCA GAA GCC-3′. Before each round of selection and binding experiments, the ssDNA library and aptamer pools were denatured by heating for 5 min at 95°C in Dulbecco’s phosphate buffered saline with CaCl_2_ and MgCl_2_ (DPBS) (Cat. # D8662, Sigma-Aldrich, ON, Canada), and then renatured on ice for 10 min. The selection process consisted of 10 rounds, starting with one round of positive selection against *Cryptosporidium parvum* oocysts, followed by six rounds of alternating negative and positive selection, and then another three rounds of positive selection. Negative selection was carried out against cysts of the protozoan parasite *Giardia duodenalis* (Waterborne, Inc., LA, USA) to ensure that the selected aptamers maintained high specificity for the target, *C*. *parvum*.

Prior to selection experiments, 3x10^5^ *C*. *parvum* oocysts (Waterborne, Inc., LA, USA) were washed twice in DPBS at 3,500 x g for 5 min and resuspended in DPBS containing 5mg/mL yeast tRNA (Cat. # 55714, EMD Millipore) and 1mg/mL bovine serum albumen (Cat. # B9000, New England BioLabs, MA, USA), and used for each round of selection. In the first round, oocysts were incubated in 100 μL of DPBS containing 1 μM of ssDNA library for 30 min at 25°C and then centrifuged at 14,000 x g for 10 min at 15°C, to remove unbound aptamers, followed by rinsing twice with DPBS. Low binding microcentrifuge 1.7 mL tubes (VWR, Cat. # 3207, Corning Life Sciences, NY, USA) were used in each round of selection.

*C*. *parvum* oocysts were resuspended in 20 μL of 1xTris-EDTA, buffer pH 8.0 (TE buffer solution, Cat. # T9285, Sigma-Aldrich, ON, Canada) and heated for 10 min at 95°C to release the aptamers bound to the oocysts. After the denaturing step, unbound oocysts were removed by centrifugation at 14,000 x g for 15 min, and the supernatant (containing the aptamers) was collected and stored at −20°C. Subsequently, oocyst-bound aptamers were amplified using both symmetric and asymmetric PCR cycles. For the symmetric PCR, 5 μL of the aptamer pool in TE buffer was mixed with 45 μL of symmetric PCR reaction mixture containing GoTaq Flexi buffer (Cat. # M891A, Promega, WI, USA), 2.5 mM MgCl_2_ (Cat. # M890A, Promega, WI, USA), 200 μM of each of the four deoxynucleotide triphosphates (dNTP) (Cat. # C1141, Promega, WI, USA), 0.5 μM of forward primer (5′-CTC CTC TGA CTG TAA CCA CG-3′), 0.5 μM reverse primer (5′-GGC TTC TGG ACT ACC TAT GC-3′) and 2.5 U of GoTaq Hot Start Polymerase (Cat. # M5001, Promega, WI, USA). The resulting amplification product was used as a template in the asymmetric PCR where 5 μL of the symmetric PCR product was mixed with 45 μL of the asymmetric PCR reaction containing the same reagents as above, with the exception that a 1 μM forward FAM primer (5′-56-FAM-CTC CTC TGA CTG TAA CCA CG-3′) and a 0.05 μM reverse primer were used. All PCR amplifications were performed using the following temperature cycle program: preheating for 2 min at 95°C, followed by 25 cycles for symmetric PCR, and 20 cycles for asymmetric PCR, of denaturing for 30 sec, at 95°C, annealing for 15 sec at 66°C, and extension for 15 sec at 72°C. The final cycle was followed by an extension step of 1 min at 72°C, and a hold at 4°C.

Fluorescently labeled ssDNA was separated from the PCR mixture, primers, and dNTPs, with 30 kDa cut-off filters (Nanosep, PALL) by centrifugation at 3,800 x g for 15 min at 15°C and washing three times with 200 μL of DPBS buffer. The quality and band intensity of an individual aptamer pool were examined by electrophoresis on 3% agarose gels containing GelRed (5 mL/100 mL) (Biotium, Inc., CA, USA). Gels were run for 35 min at 120V with 1x TE buffer (10mM Tris-HCl, 1mM EDTA, pH 8.0). Subsequently, the purified aptamer pool was diluted in 100 μL of DPBS, and its concentrations was measured by a BioDrop μLITE UV/Visible spectrophotometer (Montreal Biotech Inc., QC, Canada), and then stored at −20°C before continuation with the next round. Finally, 300 nM of the aptamer pool was utilized for the next round of selection following the same procedure.

### Flow Cytometric Binding Analysis of Pools

The affinities of all aptamer pools toward *C*. *parvum* oocysts were measured using an FC-500 flow cytometer (Beckman Coulter Inc., USA) in a similar way as described elsewhere [13]. *C*. *parvum* oocysts (3x10^5^) were preincubated for 15 min at room temperature with 5 mg/mL of yeast tRNA, which acted as a masking single-stranded nucleic acid to suppress non-specific binding of aptamers. Oocysts were then incubated with 300 nM purified 56-FAM labeled aptamer pool in 250 μL DPBS buffer for 30 min at 25°C. Control experiments were performed using the fluorescently labeled ssDNA library in DPBS. Subsequently, each sample was washed once with 250 μL of DPBS and centrifuged at 14,000 x g for 10 min to remove unbound DNA, resuspended in 500 μL of DPBS, and subjected to the flow cytometric analysis.

### Cloning and Sequencing of the Aptamer Pools

The aptamer pools showing the highest affinity to *C*. *parvum* oocysts, according to the flow cytometry results, were cloned. Briefly, the double-stranded DNA pool was extracted from the electrophoresis gel using the DNA gel extraction kit Axy prep (Cat. # AP-GX-50, AxyGen Biosciences, MA, USA), in order to purify the DNA from the PCR mixture. Cloning was performed according to the supplied protocol using pT7Blue Perfectly Blunt Cloning Kit (Cat. # 70189–3, Novagen, Germany). White colonies were collected into separate tubes, and left to grow overnight in 1 mL of SOC medium (Cat. # S1797, Sigma-Aldrich, ON, Canada) at 37°C, and then analyzed by PCR to ensure that the clones had the insert in the plasmids. Plasmids were extracted using a GeneJET plasmid miniprep kit (Cat. # K0502, Thermo Scientific / Fisher Scientific, Canada), according to the supplied protocol. Plasmids with the insert were amplified using an M13 forward primer (5′-GTA AAA CGA CGG CCA GT-3′) and M13 reverse primer (5′-AGC GGA TAA CAA TTT CAC ACA GG-3′), according to the following protocol. The reaction was run in a total of 50 μL, containing 200 μM of each of the four deoxynucleoside triphosphates (dNTP’s), 0.5 μM each of forward M13 primer and reverse M13 primer, 2.5 mM MgCl_2_, 0.2 U KAPA 2G Robust Hot Start DNA polymerase, and 1% PCR buffer A; 5 μL of the plasmid was used as template in the PCR reaction. The temperature cycling conditions were as follows: hot start at 950°C for 5 min, followed by 20 cycles of denaturing for 30 sec at 95°C, annealing for 15 sec at 58°C, extension for 10 sec at 72°C, and a hold at 4°C. The unpurified PCR product was sequenced at McGill University and Génome Québec Innovation Centre, Montreal, QC, Canada. All synthetic aptamer clones were purchased from Integrated DNA Technologies (CA, USA).

### Aptasensor Preparation

Prior to experiments, the gold nanoparticle-modified screen-printed carbon electrode (GNPs-SPCE) (DRP-110, L33xW10xH0.5, Dropsens, Spain) was washed thoroughly with deionized nuclease-free water then dried with N_2_. All nucleic acids were heated at 60°C for 5 min prior to use in order to prevent aggregation. This was followed by reduction of the thiol-modified primer, 5'-/5ThioMC6-D/GGC TTC TGG ACT ACC TAT GC-3', modified at the 5' position with a 6-hydroxyhexyl disulfide group. Briefly, 2 μL of 100 μM primer were mixed with 4 μL of 10 mM tris-(2-carboxyethyl) phosphine (TCEP) (Cat. # 646547, Sigma-Aldrich, ON, Canada) and incubated for 1 h at room temperature. The role of TCEP is to reduce the disulfide bond of the modified primer since it can selectively reduce even the most stable water-soluble alkyl disulfides over a wide pH range [14]. Afterward, aptamer denaturation was carried out by heating for 5 min at 95°C followed by snap-cooling on ice for 10 min. Subsequently, 2 μM of the reduced primer were incubated with 2 μM of the denatured aptamer for 1 h at room temp to form a DNA hybrid. Finally, the electrode was incubated overnight in a humidity chamber, with 100 nM of the hybrid in DPBS. Prior to use, the electrode was incubated with 0.1 mM 2-mercaptoethanol for 30 min to reduce the background oxygen contributions and nonspecific interactions between the probe and the gold surface and allow the probe to adopt an upright position.

### Fruit Sample Processing

Twenty-five gram samples of fresh cut fruits (pineapple and mango) were weighed out in individual stomacher bags (with a capacity of at least 1 L). To each bag, 200 ml of phosphate buffered saline with 0.01% Tween 80, pH 7.4 (PBS-Tween 80) was added. Bags were placed on an orbital shaker for 10 min at 100 rpm. The entire contents of the stomacher bag were then transferred to a VWR vacuum filter (0.2 μm) assembly unit (Cat. # 97066–202, PALL), and the filtered sample was transferred into four 50 mL conical centrifuge tubes. The tubes were centrifuged at 4,000 x g for 15 min at 4°C. The supernatant was aspirated, leaving approximately 5 mL in each tube. All four pellets from each sample were pooled into one of the centrifuge tubes by vortexing and transferring the volume into the next tube. Each tube was then rinsed with a small volume of PBS-Tween 80 buffer, which was then poured into the next tube until all tubes were rinsed. This rinse buffer was then added to the pooled tube and vortexed for a few seconds to resuspend the pellet. All pooled tubes from each sample were then re-centrifuged, as described above, and the supernatants were again aspirated and discarded. Two hundred μL of pectinase was added, and each tube was centrifuged at 5,000 x g for 30 min at 4°C. Each pellet was transferred by micropipette to a labeled 1.7 mL microcentrifuge tube, and the original conical tube was rinsed with 500 μL of PBS-Tween 80 buffer. Microcentrifuge tubes were centrifuged at 10,000 x g for 10 min, and the supernatants were aspirated and discarded. Five-hundred μL of PBS-Tween 80 buffer was added to each tube and the pellets were resuspended by vortexing. Aliquots containing 0, 300 or 700 *C*. *parvum* oocysts in 50 μL of DPBS were added to mango and pineapple concentrates and left overnight. Oocyst-spiked concentrates were then used for selectivity and specificity of the developed aptasensor and electrochemical analyses.

### Electrochemical Measurements

SWV was performed using an electrochemical analyzer (CH Instruments 660D, TX, USA) connected to a computer. All measurements were carried out at room temperature in an enclosed and grounded Faraday cage. A conventional three-electrode configuration printed on a ceramic substrate; including an aptamer-modified GNPs-SPCE electrode as the working electrode, a carbon counter electrode, and a silver pseudo-reference electrode. A three-electric contacts edge connector (Dropsens, Spain) was used to connect the screen-printed electrode with the potentiostat. The open-circuit or rest-potential of the system was measured prior to all electrochemical experiments to prevent sudden potential-related changes in the self-assembled monolayer. SWV measurements were carried out in the range of ‒400 to 800 mV with a step potential of 4 mV, amplitude of 5 mV and frequency of 10 Hz. Electrochemical measurements were performed in phosphate buffered saline (PBS) (pH 7.4), containing 2.5 mM of K_4_[Fe(CN)_6_] and 2.5 mM of K_3_[Fe(CN)_6_]. All measurements were repeated a minimum of three times with separate electrodes to obtain statistically meaningful results.

## Results and Discussion

### Selection of Aptamer Pools to *Cryptosporidium*

**Fig 2:**
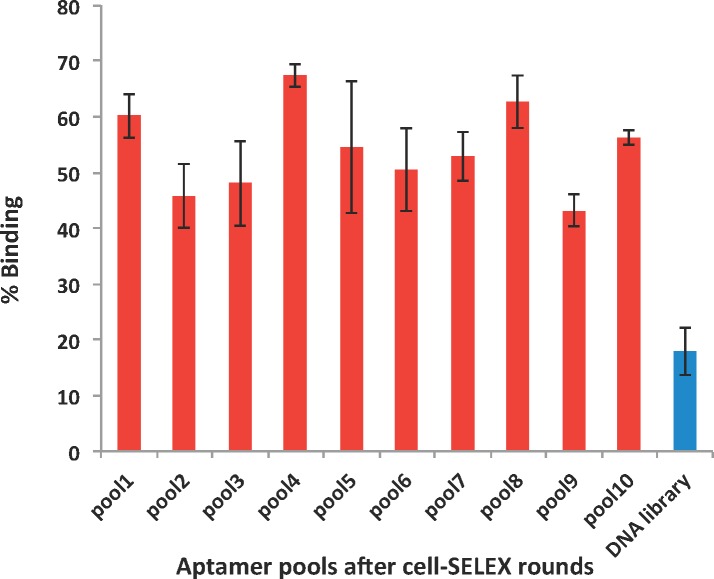
Flow cytometric analysis of the binding affinities between 3 x 10^5^ *C*. *parvum* oocysts and 300nM 56-FAM labeled aptamer pools. A control experiment was performed using the native DNA library instead of aptamer pools.

A single-stranded (ss) DNA library containing a 40-nt random region flanked by two 20-nt primer binding regions was screened for *Cryptosporidium parvum* oocyst binders using cell-SELEX, an *in vitro* evolution of nucleic acid binders to whole cells, bacteria, and viruses [15, 16]. A total of 10 rounds of selection against oocysts were performed. Briefly, each round of the selection consisted of five consecutive steps: (1) incubation of the ssDNA library with *Cryptosporidium* oocysts, (2) partitioning of unbound DNA from oocysts, (3) extraction of bound DNA sequences, (4) DNA amplification by symmetric and asymmetric PCR, and (5) purification of aptamers from PCR products. To monitor the enrichment, fluorescently labeled pools of aptamers were incubated with *Cryptosporidium* oocysts and analyzed by flow cytometry (Fig 2). The fluorescently-labeled DNA library was used as a negative control. The first, fourth and eighth pools, exhibiting the highest binding affinity to *Cryptosporidium* oocysts, were chosen for negative selection against *Giardia duodenalis* cysts to increase aptamer specificity. A small improvement in the binding affinity was also observed after these three rounds of negative selection. The first, fourth and eighth aptamer pools were cloned in *Escherichia coli*, and 14 bacterial colonies containing the aptamer insert were found to be potential *Cryptosporidium* aptamer candidates out of 40 clones. All 14 aptamers were then sequenced (Table 1), and used in the development of aptasensors and electrochemical analyses.

**Table 1:** DNA sequences of clones isolated from the 1^st^, 4^th^ and 8^th^ pools.

Clones	DNA Sequences
R1–4	F-AGA TTG CGG ATT GCC CAC GTG GAA AGT GAT TTG TTC GTC CG-cR
R4–1	F-TCT TGG GGC AGG CAT GAG GTG TGG CAG AGG TAA GGG ATA A-cR
R4–3	F-CAC ACA AAC TGA ATT CTC AGG ATG TGG TGA TGG TTT GCA T-cR
R4–6	F-GTG GTC CCG CAA AAT GCA CGA CGA GTC TTG CTT CTG ATC T-cR
R4–8	F-CTA GGT CAC GCT TAG GAT GAA TAA CGC CCT CTT GGT TAC A-cR
R4–9	F-CTC TGT GGC GCT TGG GAT GAT CAA CGG CCT CTT GGT TAC G-cR
R4–10	F-CAC TGC ATC CTC ACT ACC GAG CCC ATC ATC GCA CGG ATC GC-cR
R4–11	F-CGA ACT AAA GGG CGG AGT CCA GAC AGA AGT GGT TGT AGC T-cR
R4–12	F-ATT GTG ACT GTG AAG GTC CAG ATT GGG CAA TCC GTT GTA A-cR
R4–14	F-CGG TAC CGG CCT ATT CAT ACT TGA AAC CTG CAC TCT TAA T-cR
R8–2	F-TCT TCC GTA GCG TCA GTG TAG TGC CTC AAA TCG CAA TGT-cR
R8–2B	F-ACA GGA GTA GGC GTT AAC ATA GGG CCG TGT CGG TTG TCA G-cR
R8–5	F-CCC CAG TGA CGG GTA GCA GAG CGT CCA CAG TTT TCC TGT AT-cR
R8–6	F-CCC CAG AGG GAT CGG TAG GTG GGA CTG TAG CGT TGG ACC G-cR

Where F is the forward PCR primer (CTCCTCTGACTGTAACCACG) and cR is the reverse-complement of the reverse PCR primer (GCATAGGTAGTCCAGAAGCC).

### Affinity Analyses of Aptamer Clones

**Fig 3:**
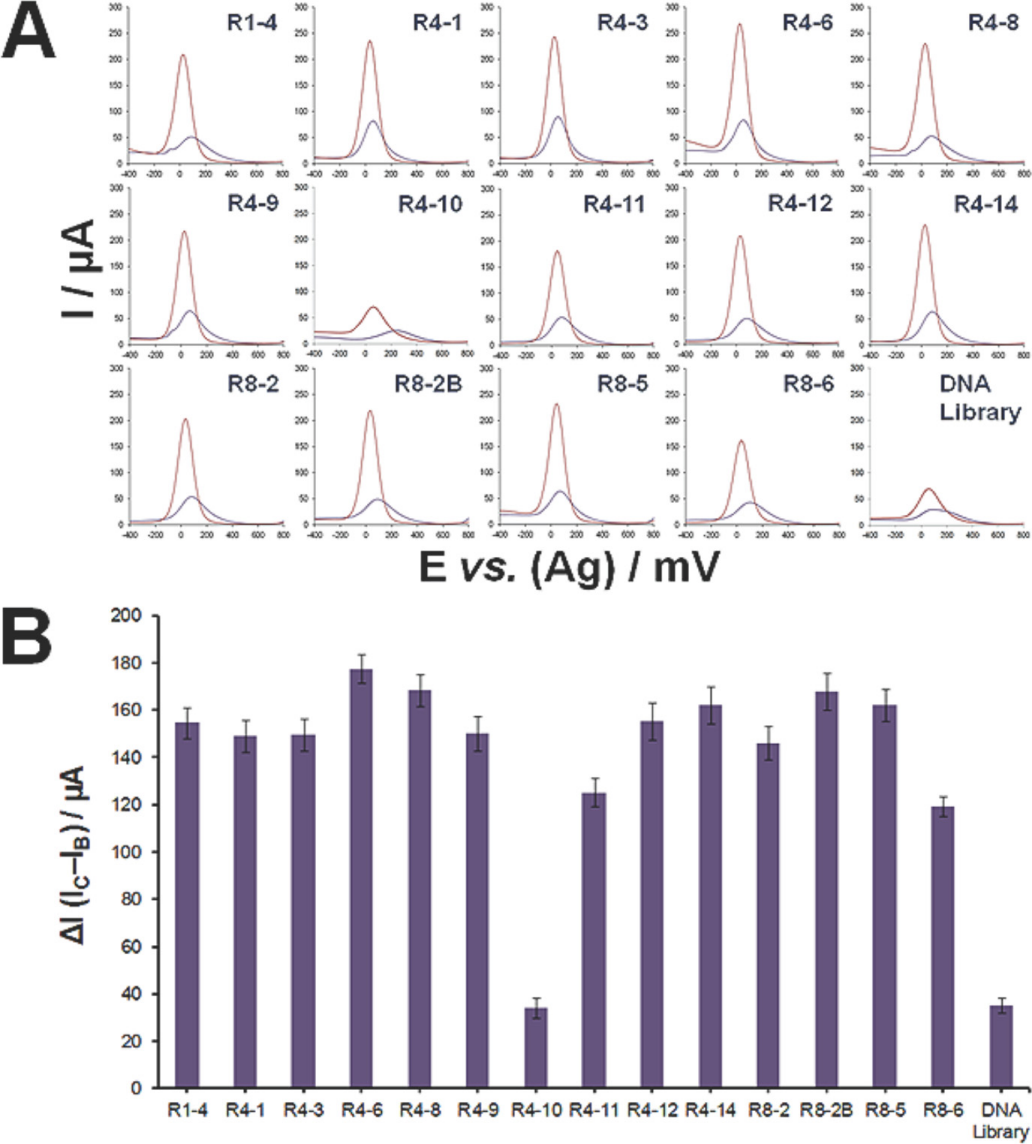
Affinity analyses of aptamer clones by square wave voltammetry. (A) Square wave voltammograms of developed aptasensors based on 14 aptamer sequences (R1–4 → R8–6) obtained before (violet curve) and after binding of 3,000 *Cryptosporidium parvum* oocysts (pink curve), whereas a control experiment is performed using an aptasensor based on the ssDNA library. All measurements were carried out after incubating the developed aptasensors with the oocysts in DPBS for 1 h at 25°C. Square wave voltammograms were carried out in the range of-400 to 800 mV with a step potential of 4 mV, amplitude of 5 mV, and frequency of 10 Hz. Electrochemical measurements were performed in PBS (pH 7.4), containing 2.5 mM of K_4_[Fe(CN)_6_] and 2.5 mM of K_3_[Fe(CN)_6_]. (**B)** Plot of the aptamer sequence *vs*. the change in current intensity (ΔI) obtained after incubation of the developed respective aptasensors with 3,000 oocysts.

A simple and inexpensive electrochemical aptasensor was used for screening 14 aptamer clones, one at a time, for *Cryptosporidium* oocysts (3,000 oocysts in 30 μL). This was carried out by incubating the aptasensors with the oocysts in DPBS for 1 h at 25°C, as shown in Fig 3A. A control experiment was performed using the ssDNA library as a non-specific detection probe instead of aptamers. To ensure accuracy, square wave voltammetry (SWV) measurements were performed before and after capturing the oocysts to guard against any possible variations in the baseline current intensity that might be caused by the different conformations adopted by aptamers on the electrode surface, thereby affecting the penetration of ions through the formed monolayer. The current intensity baseline, *I*_*B*_, and after *C*. *parvum* oocyst binding, *I*_*C*_, were measured and ∆I, was calculated from the formula: ∆I *= I*_*C*_ *− I*_*B*_. The value of ∆I is indicative of the affinity of the respective aptamer to the oocyst. As can be seen in Fig 3B, the R4–6 aptamer exhibited the highest binding affinity to the oocysts as evidenced by a large *∆I* value (177.5 ± 6.1 μA).

### Limit of Detection of Aptasensor

**Fig 4:**
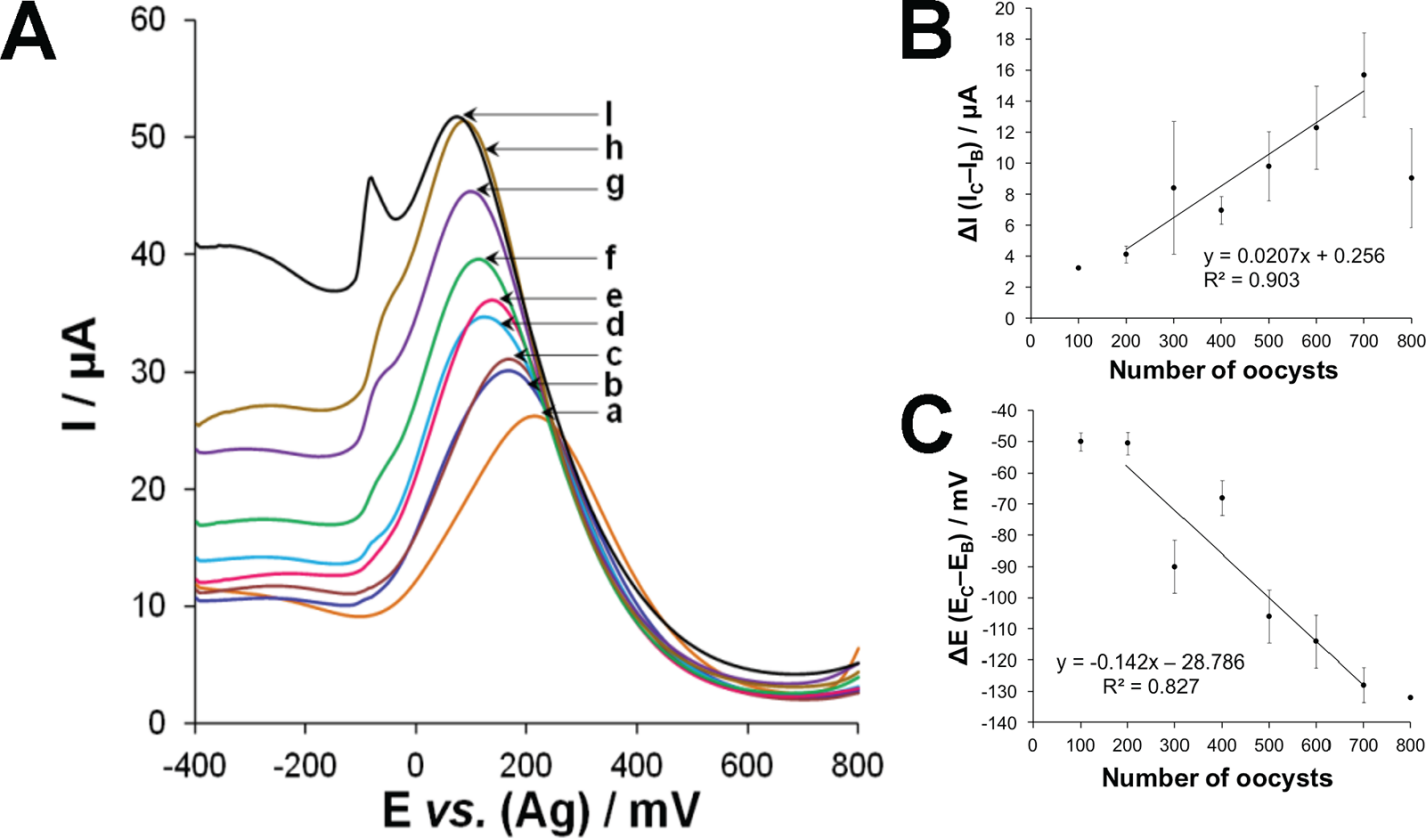
Limit of detection of the aptasensor. (A) Square wave voltammograms obtained after incubating the R4–6 aptamer-based sensors with (*a*) 0, (*b*) 100, (*c*) 200, (*d*) 300, (*e*) 400, (*f*) 500, (*g*) 600, (*h*) 700, and (*i*) 800 *Cryptosporidium parvum* oocysts. (B) Calibration plot of the change in current intensity (ΔI) *vs*. number of oocysts. (C) Calibration plot of the change in potential (ΔE) *vs*. number of oocysts.

Prior to titration experiments, aliquots containing different numbers of oocysts (0, 100, 200, 300, 400, 500, 600, 700, or 800 oocysts) in 30 μL of DPBS were incubated with the R4–6 aptamer-based sensor at 25°C for 1 h. SWV was performed and it was observed that the binding between the oocysts and immobilized aptamer caused an increase in the current intensity and a cathodic shift, as shown in Fig 4A. The measurement time was less than 10 sec. This could be attributed to a conformational change in the aptamers after binding to the oocysts and surface restructuring, which allows the external redox probe to penetrate more freely to the electrode (25, 26). Hence, the modulation of the electrochemical signal was recorded as a function of the current intensity (I) and peak potential (E). As shown in Fig 4B, the ∆I value increased linearly with an increase in the number of oocysts, in the range from 200 to 700 oocysts, with the regression equation of *y* = 0.0207 *x* + 0.2558 (*R* ^*2*^ = 0.9034), where *y* is the ∆I value in µA and *x* is the number of oocysts. At greater than 800 oocysts, the response became nonlinear indicating the saturation of the surface. As shown in Fig 4C, the ∆E value, calculated from the formula: ∆E *= E*_*C*_ *− E*_B,_ decreased linearly with an increasing number of oocysts (i.e., 200 to 700), with the regression equation of *y* = –0.1421 *x*—28.786 (*R* ^*2*^ = 0.8271), where *y* is the ∆E value in mV and *x* is the number of oocysts. The relative standard deviation values were between 4.4 and 9.4%, and the limit of detection was approximately 100 oocysts. The LOD was estimated from the formula 3(*S*_*b*_/*m*), where *S*_*b*_ is the standard deviation of the measurement signal of 0 oocyst and *m* is the slope of the analytical curve in the linear region. The median infectious dose for *C*. *parvum* ranges from less than 10 to over 1,000 oocysts based on human volunteer studies [2]. The use of DNA aptamers, therefore, may provide the level of detection, which is sensitive enough to identify potentially infectious concentrations of *C*. *parvum* oocysts on fresh produce. We suggest that both analytical signals (∆I and ∆E) provide a two-dimensional assay for the *C*. *parvum* oocysts. It should be noted that electrochemical analysis of the oocysts required a vigorous de-aeration of the measurement buffer. This was carried out using pure N_2_ gas for 30 min prior to analysis.

### Selectivity and Specificity of Aptasensor

**Fig 5:**
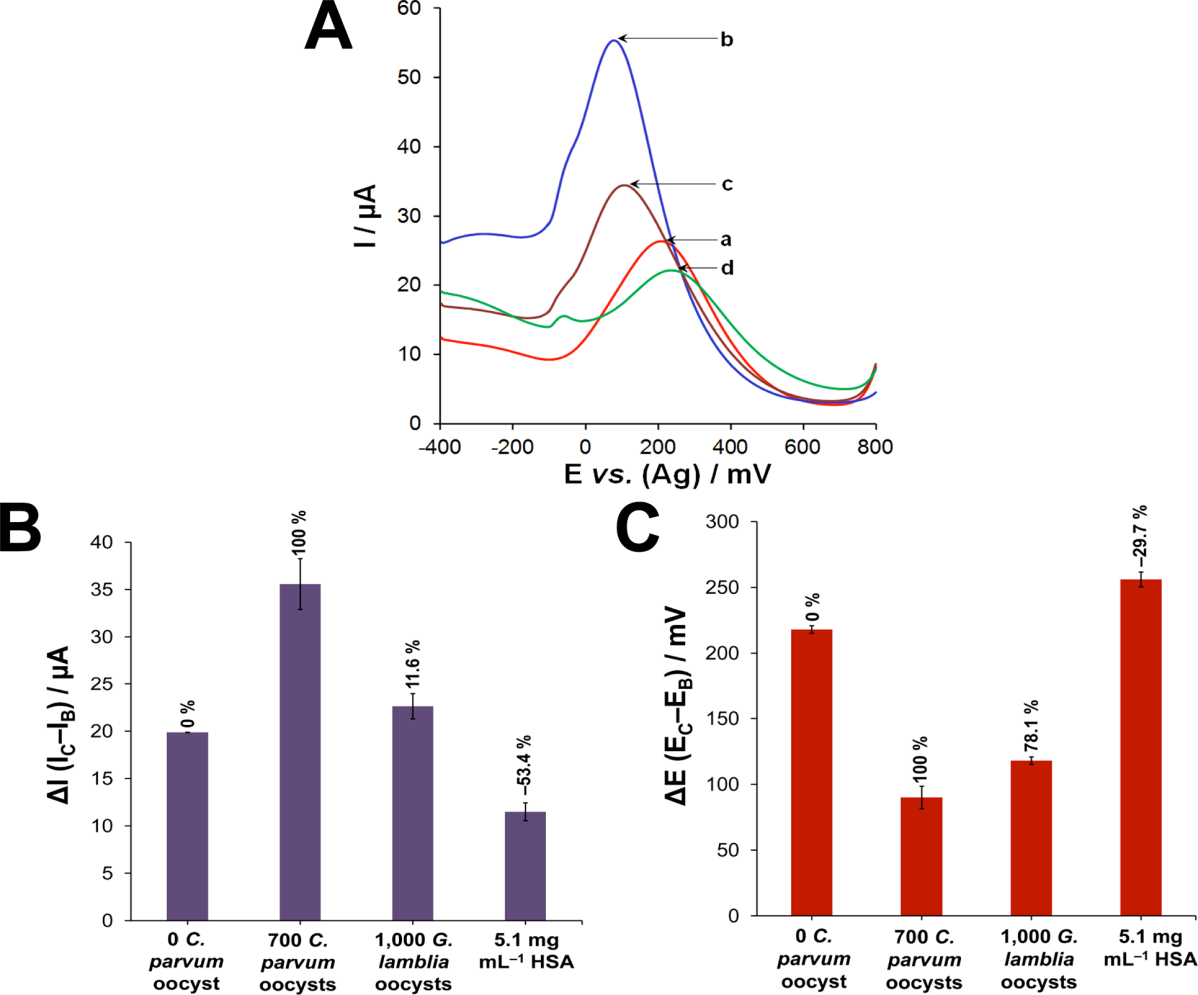
Selectivity and specificity of the aptasensor. (A) Square wave voltammograms of the selectivity experiments performed by incubating the R4–6 aptamer-based sensor with (*a*) buffer alone, (*b*) 700 *C*. *parvum* oocysts, and (*c*) 1,000 *G*. *duodenalis* cysts, and (*d*) 5.1 mg/mL HSA. (B) Plot of ΔI and (C) ΔE *vs*. the tested target.

**Fig 6:**
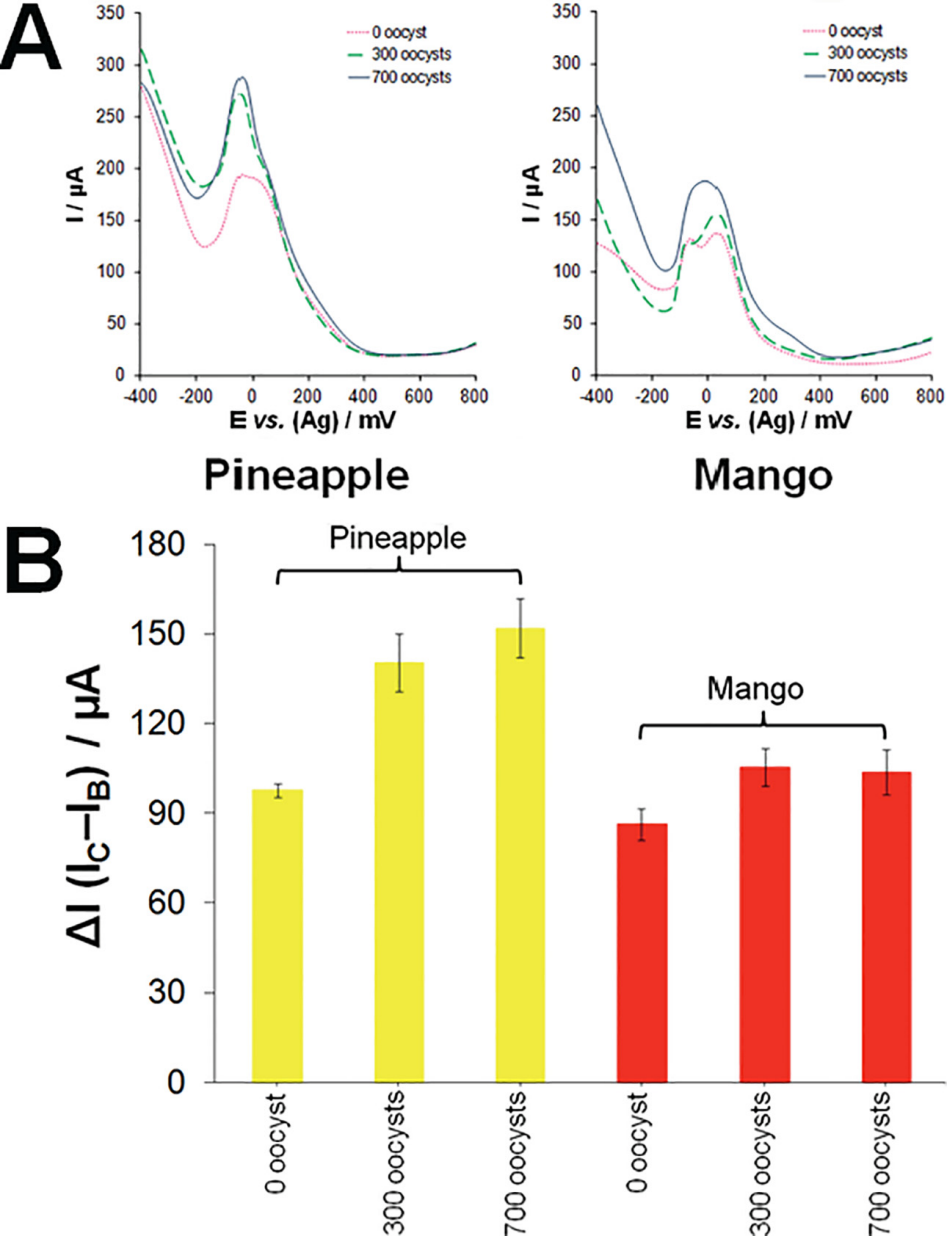
Detection of *C*. *parvum* in fruit concentrates. (A) Square wave voltammograms of the selectivity experiments performed by incubating the R4–6 aptamer-based sensor with (*a*) buffer alone, (*b*) 300 *Cryptosporidium parvum* oocysts, and (*c*) 700 *C*. *parvum* oocysts, in pineapple and mango concentrates. (B) Plot of ΔI *vs*. the tested target. All measurements were repeated three times with separate electrodes (p < 0.005).

The aptasensor’s ability to distinguish between *C*. *parvum* and other parasites, e.g., *Giardia duodenalis* was confirmed by SWV experiments, as shown in Fig 5. It was observed that incubation of the R4–6 aptamer-based sensor with 1,000 cysts of *G*. *duodenalis* resulted in an 11.6% increase in the ∆I value (22.6 ± 1.3 μA) and a 78.1% decrease in the ∆E value (118 ± 2.8 mV). Percentages were obtained with reference to the incubation with buffer alone (0%, 19.9 ± 0.02 μA, 218 ± 2.8 mV) and 700 oocysts of *C*. *parvum* (100%, 35.6 ± 2.7 μA, 90 ± 8.5 mV). The specificity of the sensor was also tested using 5.1 mg/mL human serum albumin, which caused a 53.4% decrease in the ∆I value (11.5 ± 0.9 μA) and 29.7% increase in the ∆E value (256 ± 5.7 mV). The results show that incubation with serum proteins causes a reduction in the current intensity and anodic shift as a result of fouling of the sensor surface. However, due to the signal-ON nature of the developed sensor, incubation with the target causes an opposite signal, thus leading to an increase in current intensity and cathodic shift. Furthermore, pineapple and mango concentrates were spiked with 0, 300 and 700 oocysts of *C*. *parvum* and analyzed using the developed electrochemical aptasensor. This was carried out by mixing equal volumes of the fruit concentrate and oocysts in DPBS in order to control the pH and prevent the degradation of the immobilized aptamers on the sensor surface. A control experiment was performed using equal volumes of the concentrate and DPBS only. As can be seen in Fig 6A and 6B, the aptasensor was capable of detecting the oocysts in the fruit concentrates as evidenced by the relative increase in the current intensity, regardless of the non-specific adsorption of the concentrate content onto the sensor surface. These results clearly signify the merit of the developed signal-ON sensor since the generated signal caused by the presence of the target oocysts outweighed the reduction of current caused by the non-specific adsorption of the matrix content.

## Conclusions

In the present study, ten rounds of selection led to the recognition of a number of promising aptamers with very high affinity for *C*. *parvum* oocysts. Using flow cytometry, these aptamers were found to bind to *C*. *parvum* oocysts with an affinity in the low nanomolar range. The present study is the first to report the effectiveness of aptamers, as alternatives to monoclonal antibodies, for the detection of *Cryptosporidium* oocysts on fresh produce.

The high sensitivity and specificity of the aptamers selected in the present study suggest that they may be very useful in detecting the presence of *C*. *parvum* oocysts on foods in both outbreak investigations and surveillance studies, as well as in routine water testing. Specifically, the low oocyst detection limit of the aptamers selected in the present study, and high level of discrimination against background currents, suggest that this novel method may be a useful alternative to immunofluorescence microscopy. Furthermore, since aptamers are single-stranded oligonucleotides, they are considerably less expensive to produce than the monoclonal antibodies used in conventional testing, and have much longer shelf life. Work is currently underway in our laboratory to validate the use of these aptamers in the detection of *C*. *parvum* oocysts on fresh produce against the conventional method of immunofluorescence microscopy, using commercial monoclonal antibodies. We are also developing an enzyme-linked immunosorbent assay (ELISA), targeting the aptamer-oocyst complex, to simplify and accelerate the detection procedure. Specificity testing is also underway to determine whether the selected aptamers are genus specific, i.e., will target all species of *Cryptosporidium*, or only *C*. *parvum*. A determination of the ability of these aptamers to distinguish amongst different genotypes or sub-genotypes of *Cryptosporidium* spp. will also be of great interest, and will have considerable implications in terms of source-tracking and risk management.
